# Supplementary material for: Micro-costing analysis of a combination intervention for improved mental health and HIV risk behaviors among school-going adolescent girls in Uganda
Source: PLOS Ment Health. 2024 Dec 6;1(7):e0000201. doi: 10.1371/journal.pmen.0000201 (PMC12499882; doi:10.1371/journal.pmen.0000201)
Supplement: S1 Text — (DOCX) [file pmen.0000201.s001.docx]

S1 Text. Additional description of the costing methods

1. Valuation of program staff time

The Suubi4Her program staff were involved with the implementation of several other programs during the intervention period. In order to capture their effort dedicated to Suubi4Her, we regularly reviewed the study protocols and administrative records of the program to identify the days when program activities took place over the intervention period. This information was then corroborated during periodic interviews by key program staff during the intervention period. The interviews with key program staff indicated that program staff spent, on average, 40% of their time on Suubi4Her, and, further, they spent, on average, 80% of their time on program activities while the remaining 20% was devoted to research activities. To value staff time, we used an average hourly salary rate of 8,471 UGX, which was estimated based on average annual gross wage rate of 16,263,578 UGX.

1. Valuation of donated time by schoolteachers

Donated time includes time spent by teachers for mobilizing families to participate in program activities, and time spent by bank officials for the opening of youth development accounts (YDAs). Hourly rate was calculated as 2,163 UGX per hour for teachers and 2,500 UGX per hour for bank officials, based on their respective average gross annual income of 4,152,960 UGX and 5,760,000 UGX. Time spent on mobilization activities was determined based on interviews with teachers conducted by key program staff. Time spent on account opening was determined according to the study protocol and the administrative records of the program which was further corroborated by key program staff. The total cost of each activity was derived by multiplying the unit cost per hour by the number of hours spent on that activity.

1. Participation and facilitation incentives

Families received monetary incentive for their participation (8,000 UGX per day) in program-related activities to compensate for their time and travel costs. The total cost was derived based on the number of families participating across all program activities over the trial period.

Teachers received monetary incentive (15,000 UGX per day) to cover time and cost of phone calls they made to mobilize families for program-related activities at schools. The total cost was derived based on the number of days teachers mobilized families to attend scheduled program activities, such as FLT sessions, IGM workshops, and MFG sessions, over the trial period.

Parent peers (PPs) and community health workers (CHWs) received monetary incentives for participating in MFG trainings (45,000 UGX per day) and for facilitating MFG sessions with families (60,000 UGX per day) to compensate for their time and travel costs. The total cost was derived based on the number of days the trainings were held and the number of MFG sessions that were facilitated by PPs and CHWs over the trial period.

Teachers and stakeholders received monetary incentives for their participation (50,000 UGX per day) in stakeholder and dissemination meetings to cover their travel costs. The total was derived based on the number of meetings held and the number of people who attended each meeting over the trial period.

1. Valuation of donated space

Donated space includes classroom space used to deliver program-related activities at participating schools. The unit cost per day was based on approximate average market cost of renting a similar size venue for a day (50,000 UGX per day) which was confirmed and approved by school leadership. We multiplied this unit cost by the number of days classroom space was used to deliver program-related activities by study arm.
