# Supplementary material for: Micro-costing analysis of a combination intervention for improved mental health and HIV risk behaviors among school-going adolescent girls in Uganda
Source: PLOS Ment Health. 2024 Dec 6;1(7):e0000201. doi: 10.1371/journal.pmen.0000201 (PMC12499882; doi:10.1371/journal.pmen.0000201)
Supplement: Attachment — Submitted filename: Rebuttal Letter_Suubi4Her.docx [file pmen.0000201.s003.docx]

10/11/2024

Dear Editor,

Thank you for the opportunity to revise and improve the manuscript. Please see our response to the comments below.

Best regards,

Yesim Tozan

Journal Requirements:

1. Please include a complete copy of PLOS’ questionnaire on inclusivity in global research in your revised manuscript. Our policy for research in this area aims to improve transparency in the reporting of research performed outside of researchers’ own country or community. The policy applies to researchers who have travelled to a different country to conduct research, research with Indigenous populations or their lands, and research on cultural artefacts. The questionnaire can also be requested at the journal’s discretion for any other submissions, even if these conditions are not met.  Please find more information on the policy and a link to download a blank copy of the questionnaire here: [https://journals.plos.org/plosmentalhealth/s/best-practices-in-research-reporting](https://urldefense.proofpoint.com/v2/url?u=https-3A__journals.plos.org_plosmentalhealth_s_best-2Dpractices-2Din-2Dresearch-2Dreporting&d=DwQBaQ&c=slrrB7dE8n7gBJbeO0g-IQ&r=XGzUN6S7CV-sOjf7f4TPQQ&m=uB59FUo4gy3OSja3oxcktEvVpn4OT_07-ldyR2hrYzmjtFFIwM_50sAfM3r9b08w&s=v8lhePh71VBG4BKsP72bkv4s40HDz8gd3OT1Or-iIbU&e=). Please upload a completed version of your questionnaire as Supporting Information when you resubmit your manuscript.

-> A completed questionnaire is included with the resubmission.

2. We noticed you have some minor occurrence of overlapping text with the following previous publication(s), which needs to be addressed:

[https://www.ajtmh.org/view/journals/tpmd/106/4/article-p1078.xml](https://urldefense.proofpoint.com/v2/url?u=https-3A__www.ajtmh.org_view_journals_tpmd_106_4_article-2Dp1078.xml&d=DwQBaQ&c=slrrB7dE8n7gBJbeO0g-IQ&r=XGzUN6S7CV-sOjf7f4TPQQ&m=uB59FUo4gy3OSja3oxcktEvVpn4OT_07-ldyR2hrYzmjtFFIwM_50sAfM3r9b08w&s=iqNPub0UQuVacfMcXmIoYPyGoIxjpvB-FrYN5zbS4uA&e=)
[https://bmcpublichealth.biomedcentral.com/articles/10.1186/s12889-023-15083-2](https://urldefense.proofpoint.com/v2/url?u=https-3A__bmcpublichealth.biomedcentral.com_articles_10.1186_s12889-2D023-2D15083-2D2&d=DwQBaQ&c=slrrB7dE8n7gBJbeO0g-IQ&r=XGzUN6S7CV-sOjf7f4TPQQ&m=uB59FUo4gy3OSja3oxcktEvVpn4OT_07-ldyR2hrYzmjtFFIwM_50sAfM3r9b08w&s=l4ov2GZ_bmfB8VbSBM4eszyReBo0HvC4FFiPBv9tnqw&e=)

In your revision ensure you cite all your sources (including your own works), and quote or rephrase any duplicated text outside the methods section. Further consideration is dependent on these concerns being addressed.

-> Thank you for your feedback. We have thoroughly reviewed the manuscript and made the necessary revisions to ensure proper citation, rephrasing, and addressing any duplicated text outside the methods section.

Additional Editor Comments (if provided):

1) References in Introduction and Discussion: Please review and update the references throughout your manuscript. It is crucial that these references accurately support the claims you make. For instance, the data cited on the current HIV epidemic (Lines 56-58) rely on a reference from 2017. These figures are outdated. Please cite more recent data, for example the 2023 Spectrum estimates available at [https://aidsinfo.unaids.org/](https://urldefense.proofpoint.com/v2/url?u=https-3A__aidsinfo.unaids.org_&d=DwQBaQ&c=slrrB7dE8n7gBJbeO0g-IQ&r=XGzUN6S7CV-sOjf7f4TPQQ&m=uB59FUo4gy3OSja3oxcktEvVpn4OT_07-ldyR2hrYzmjtFFIwM_50sAfM3r9b08w&s=BxN92qg0fu8gnD9RuUs0iEc54VOTJcGml53zjomxaCw&e=).

->Thank you for your feedback and for pointing out the need to update the references in the Introduction and Discussion sections. we have carefully reviewed the manuscript and made the necessary revisions to ensure that all references are current and accurately support the claims made throughout the text. Specifically, we have replaced the outdated data on the HIV epidemic (Lines 56-58) with the most recent figures, including the 2023 Spectrum estimates as suggested.

2) First Sentence of the Discussion: The opening sentence of the discussion section needs reconsideration. While acknowledging that there are significant gaps in HIV care across sub-Saharan Africa, it is incorrect to state that HIV services are non-existent. Most countries in the region have established robust pediatric HIV programs. Please revise this statement to reflect a more accurate depiction of the HIV services landscape.

-> Thank you for our feedback. We have now revised this sentence and updated the references to support the claims made in this sentence.

Reviewers' comments:

Reviewer's Responses to Questions

**Comments to the Author**

1. Does this manuscript meet PLOS Mental Health’s [publication criteria](https://urldefense.proofpoint.com/v2/url?u=https-3A__journals.plos.org_mentalhealth_s_criteria-2Dfor-2Dpublication&d=DwMBaQ&c=slrrB7dE8n7gBJbeO0g-IQ&r=XGzUN6S7CV-sOjf7f4TPQQ&m=uB59FUo4gy3OSja3oxcktEvVpn4OT_07-ldyR2hrYzmjtFFIwM_50sAfM3r9b08w&s=CGKz1N2AtBXjwuEhmNJVghTOY4rCTutJ-zwFgNfxjek&e=)? Is the manuscript technically sound, and do the data support the conclusions? The manuscript must describe methodologically and ethically rigorous research with conclusions that are appropriately drawn based on the data presented.

Reviewer #1: Yes

2. Has the statistical analysis been performed appropriately and rigorously?

Reviewer #1: Yes

3. Have the authors made all data underlying the findings in their manuscript fully available (please refer to the Data Availability Statement at the start of the manuscript PDF file)?

The [PLOS Data policy](https://urldefense.proofpoint.com/v2/url?u=https-3A__journals.plos.org_mentalhealth_s_data-2Davailability&d=DwMBaQ&c=slrrB7dE8n7gBJbeO0g-IQ&r=XGzUN6S7CV-sOjf7f4TPQQ&m=uB59FUo4gy3OSja3oxcktEvVpn4OT_07-ldyR2hrYzmjtFFIwM_50sAfM3r9b08w&s=fIKJ6siBmc1y6CVgZhXrwozAm7Ignu8U9gu4YvCUZ_8&e=) requires authors to make all data underlying the findings described in their manuscript fully available without restriction, with rare exception. The data should be provided as part of the manuscript or its supporting information, or deposited to a public repository. For example, in addition to summary statistics, the data points behind means, medians and variance measures should be available. If there are restrictions on publicly sharing data—e.g. participant privacy or use of data from a third party—those must be specified.

Reviewer #1: No

### -> We have included the following Data Availability Statement in the revised manuscript:

### Data Availability Statement

All relevant data supporting the findings of this study are available upon request from the International Center for Child Health and Development (ICCHD), Uganda. Data can be obtained by contacting [ichad@wustl.edu](mailto:ichad@wustl.edu).

4. Is the manuscript presented in an intelligible fashion and written in standard English?

PLOS Mental Health does not copyedit accepted manuscripts, so the language in submitted articles must be clear, correct, and unambiguous. Any typographical or grammatical errors should be corrected at revision, so please note any specific errors here.

Reviewer #1: Yes

5. Review Comments to the Author

Please use the space provided to explain your answers to the questions above. You may also include additional comments for the author, including concerns about dual publication, research ethics, or publication ethics. (Please upload your review as an attachment if it exceeds 20,000 characters)

Reviewer #1: In this article, Tozan and colleagues present an economic analysis of the impact of the Suubi4Her combination intervention for adolescent girls in Uganda. The results are based on a randomized trial where eligible adolescents were divided into three groups: those receiving only education; those receiving also a financial support intervention; and those receiving, in addition, also a family strengthening intervention. The per-person costs for the two levels of intervention were estimated at about $500 and $800, which is comparable for other similar interventions.

I have the following comments and suggestions:

1)One major limitation is the lack of an estimate of the benefit. In the protocol at [clinicaltrials.gov](http://clinicaltrials.gov/), there is no outcome related directly to mental health – is there a plan to quantify the benefit in terms of prevented mental health related disorders etc in the future? The protocol also mentions yearly cost-effectiveness calculations (which include also the estimation of the benefits, not only costs) – have these been performed in the past years? I understand that the estimation of benefits is a complex issue (and also will need more follow-up time), but you could in the Discussion give some rough ideas of the magnitude of the burden that could be potentially preventable. This could also put the cost estimates better into context ($800 per adolescent may sound a lot, but it could well be that the consequences of the mental health problems are even much higher).

-> Thank you very much for your thoughtful feedback. The scope of this analysis focuses specifically on estimating the costs of the combination intervention, as outlined in the study protocol (please see the link to this paper below). Our primary aim was to provide context-specific cost data that reflects the resource requirements for implementing the combination intervention in a resource-limited setting like Uganda. This information is crucial for assessing the feasibility, affordability and long-term sustainability of the intervention. Additionally, the findings enable us to compare the cost of this intervention with that of other interventions targeting similar outcomes, such as mental health and HIV prevention. The primary outcomes of the Suubi4Her study include HIV risk behavior and mental health functioning, as specified in the protocol paper. The team has already published the effectiveness results in a recent paper which we have now cited in the Discussion section (Lines 740-742). We are working on a second manuscript that evaluates the cost-effectiveness of the combination intervention by combining the effectiveness data with the cost analysis results to assess its overall economic value. I am pleased to inform you that this second paper has just been submitted to a peer-reviewed journal for publication.

2) Could you describe YDA still in a simpler way? Is it a requirement that family members / relatives also contribute, e.g. if the family pays $50 then the program will also pay $50 (is this what is meant by the 1:1 ratio)? How about those whose families cannot afford any payment? Is there any upper or lower limit for the amount that is given by the program? One could argue that this intervention helps most those who are already wealthy, i.e. whose families can afford big contributions.

-> Thank you for thoughtful feedback. You are correct that the 1:1 ratio in this context means that for every unit of money contributed by the family, the program contributes an equal amount. This ensures that the family's contribution is doubled by the program's support, making it a 1:1 match. We have clarified this point in Lines 357-358. YDAs are savings accounts, designed to encourage adolescents/youth to save money and foster positive behaviors. In that sense, there is no upper or lower limit set by the program. However, we fully agree with you that the effectiveness of YDAs in improving mental health and other health outcomes and in achieving equity in low-income settings depend on how they are designed and implemented to overcome the barriers that prevent the most vulnerable youth from participating and benefiting. We believe that future research is essential to better understand these factors and to design more effective and equitable YDA programs.

3) With the payer perspective, are the costs limited to those paid externally, i.e. from the program? How about out-of-pocket costs to the families? Is it correct that the contributions of the families to YDA are not considered in the total costs?

->Thank you for your comment. We would like to clarify that the costing analysis was conducted from the ‘provider’ perspective, and as such, the contributions made by family members to the YDA accounts were hence not included in the costing analysis.

4) The use of micro-costing approach can lead to very precise estimates - does this level of precision really reflect the level of confidence we have in the results? How well are the results generalizable to other similar settings? In my understanding, the micro-costing approach also ignores the potential synergies between different programs (would it not be more reliable to just rather at the end of the project compare the total costs in all settings)?

-> Thank you for this constructive comment. The micro-costing approach is widely considered the gold standard for estimating the costs of interventions due to its ability to provide a detailed, activity-specific analysis of resource use. However, while it provides highly granular and accurate data, the generalizability of its results can be limited by the specific context in which the study is conducted. This is primarily because the cost estimates derived from a micro-costing study are often affected by local factors such as personnel costs and resource availability as well as the overall implementation setting. As a result, while the approach provides valuable insights for understanding of the cost structure of an intervention in a given context, its findings may not always be directly applicable to other settings with different economic or logistical conditions. We have already indicated this limitation in the Discussion section (Lines 1561-1565). This limitation is, however, balanced by the granular and comprehensive understanding of the inputs required for each intervention activity, which in turn allows for accurate and context-specific estimates. Further, the method highlights key cost drivers, enabling policymakers and program implementers to understand which factors contribute most to overall costs and where efficiency gains may be possible. All of this information is especially important when evaluating new or innovative interventions for which there may be no prior cost data. We have added a summary of the points made above to the Discussion section to expand our discussion and to thoroughly address your comment (Lines 1563-1568).

5) What is the rationale to use discounting in this project? I understand this is useful for future projections so that costs and benefits in the near future are given greater weight; but in the present analysis, the costs were anyway estimated retrospectively?

-> Thank you for your comment. **Discounting of costs** is considered **best practice** in health economics and is strongly recommended by established guidelines. Yes, you are correct that discounting is generally useful for future projections so that the costs and benefits in the near future are given greater weight. But more generally, **discounting i**s the recommended method to adjust and sum up costs incurred over a period of time, particularly when you are comparing costs over multiple years. In costing studies, it is recommended that the costs of an intervention or a program are adjusted to the start year of the intervention. The Suubi4Her intervention was delivered over two years. We therefore applied discounting to adjust the costs back to the start year of the intervention, sum up, and reflect the total costs in the context of the start year (2018). A common discount rate used in health economics is 3% per year, and this is the discount rate used in this analysis.

6. PLOS authors have the option to publish the peer review history of their article ([what does this mean?](https://urldefense.proofpoint.com/v2/url?u=https-3A__journals.plos.org_mentalthealth_s_editorial-2Dand-2Dpeer-2Dreview-2Dprocess-23loc-2Dpeer-2Dreview-2Dhistory&d=DwMBaQ&c=slrrB7dE8n7gBJbeO0g-IQ&r=XGzUN6S7CV-sOjf7f4TPQQ&m=uB59FUo4gy3OSja3oxcktEvVpn4OT_07-ldyR2hrYzmjtFFIwM_50sAfM3r9b08w&s=eHrCx6WKkrBhR2iP8S0IBaZsxhSo0dzCxYmNHY6ehjo&e=)). If published, this will include your full peer review and any attached files.

**Do you want your identity to be public for this peer review?** If you choose “no”, your identity will remain anonymous but your review may still be made public.

For information about this choice, including consent withdrawal, please see our [Privacy Policy](https://urldefense.proofpoint.com/v2/url?u=https-3A__www.plos.org_privacy-2Dpolicy&d=DwMBaQ&c=slrrB7dE8n7gBJbeO0g-IQ&r=XGzUN6S7CV-sOjf7f4TPQQ&m=uB59FUo4gy3OSja3oxcktEvVpn4OT_07-ldyR2hrYzmjtFFIwM_50sAfM3r9b08w&s=FyOZbKegGorkghdg6Vca2bXSvJ9lGjxPPx52wlxtsYg&e=).

Reviewer #1: No
